# Reforming Graduate Medical Education in Syria: A Strategic Framework for Post-Conflict Recovery

**DOI:** 10.1055/s-0045-1811693

**Published:** 2025-09-26

**Authors:** Oase Sbei, Amjad Rass, Abd Arrahman Alomar, Ahmed Muhbeddine, Abdulghani Sankari

**Affiliations:** 1 Department of Medicine, Wayne State University School of Medicine, Detroit, Michigan, United States; 2 Syrian American Medical Society Institute, Detroit, Michigan, United States; 3 Henry Ford Health Providence Hospital, Southfield, Michigan, United States

**Keywords:** Syria, graduate medical education, post-conflict recovery, medical training, accreditation, curriculum reform, healthcare workforce, medical education reform

## Abstract

**Background:**

More than a decade of armed conflict has devastated Syria's healthcare system, severely disrupting graduate medical education (GME) across the country. Damage to teaching hospitals, displacement of faculty, and fragmented oversight have contributed to deteriorating educational standards and a growing physician shortage. As Syria transitions into a post-conflict recovery phase, reforming its GME system is a national and global priority.

**Objective:**

This white paper aims to evaluate the current state of GME in Syria and propose a strategic framework for rebuilding a standardized, sustainable, and internationally aligned system through stakeholder engagement, data collection, and comparative analysis.

**Methods:**

In February 2025, the Syrian American Medical Society (SAMS) conducted workshops in Damascus and Aleppo involving over 45 stakeholders, including teaching hospital directors, medical educators, and diaspora physicians. A pre-workshop survey assessed program structure, oversight, curricula, evaluation methods, and infrastructure across 21 institutions. Workshop discussions were informed by a Strengths, Weaknesses, Opportunities, and Threats (SWOT) analysis and global best practices from countries including Jordan, Saudi Arabia, and the United States.

**Results:**

Findings revealed wide variability in program oversight, clinical training quality, evaluation standards, and faculty support. Only 57% of institutions reported having formal curricula, while 81% conducted some form of trainee evaluation. Common challenges included inadequate financial support, lack of standardized accreditation, insufficient faculty development, and limited research access. Recommendations from the workshops included the creation of a national accreditation council, modernization of curricula, investment in faculty training, development of centers of excellence, and integration of online education and 25 continuing medical education.

**Conclusion:**

Reforming Syria's GME system requires coordinated, multilevel efforts to implement competency-based education, establish independent regulatory bodies, and align training programs with global standards. The phased framework presented here offers actionable steps to rebuild Syria's medical education infrastructure and train a resilient health workforce capable of addressing both national and regional healthcare needs.

## Executive Summary

The prolonged conflict in Syria has severely damaged the nation's healthcare and graduate medical education (GME) infrastructure. 1 Clinical training has been disrupted, qualified faculty displaced, and program oversight fragmented. 2 To address these challenges, the Syrian American Medical Society (SAMS) conducted in-depth workshops and surveys in Damascus and Aleppo in February 2025, engaging over 45 stakeholders, including directors of teaching hospitals, medical educators, and Syrian diaspora experts. This white paper synthesizes the survey results and workshop recommendations, presenting a strategic, phased implementation plan to establish a robust, standardized, sustainable, and internationally aligned GME system. Recommendations include establishing an independent national accreditation body, modernizing curricula, strengthening faculty and resident support, investing in infrastructure, and building international partnerships. Drawing on global models from Jordan, Saudi Arabia, and the United States, this framework aligns medical education with national health priorities and global standards.

## Background and Context

Syria's prolonged conflict has left an indelible mark on its healthcare and medical education systems. 3 After more than a decade of war, the country is emerging from one of the most devastating humanitarian crises of the 21st century, with millions displaced and essential services, including healthcare, severely disrupted. 4 5 As Syria begins the recovery process, rebuilding healthcare infrastructure, restoring access to critical services, and supporting the return of health professionals are urgent national priorities. 6

Major teaching hospitals and academic institutions have sustained significant damage due to airstrikes and prolonged neglect. The displacement of qualified faculty and the emigration of thousands of medical professionals have further weakened Syria's clinical training capacity. 7 Amid this crisis, Syria now faces not only a national shortage but is also part of a global healthcare workforce crisis. According to the World Health Organization (WHO), there is a current global shortfall of over 4 million healthcare workers, a figure projected to rise to 10 million by 2030, particularly in low- and lower-middle-income countries like Syria. 8

Globally, millions still lack access to quality healthcare due to critical shortages and inequitable distribution of trained professionals, including physicians, nurses, midwives, and allied health workers. Urgent investment is required to reverse this trend. The WHO's *Global Strategy on Human Resources for Health: Workforce 2030* provides benchmarks that are especially relevant to Syria's post-conflict rebuilding efforts. These include the creation of national accreditation systems for health education by 2020 to ensure training quality; reduction of geographic disparities in workforce distribution by 2030; improved graduation rates in medical and allied health programs; and halving dependency on foreign-trained professionals by strengthening domestic capacity. The strategy also advocates for the creation of 10 million new healthcare jobs globally, particularly in underserved regions, and emphasizes the importance of sustained financial investment in training, recruitment, and retention. 8

Despite these benchmarks, Syria currently lacks a unified national framework for GME. Unlike regional counterparts such as Jordan and Saudi Arabia, Syria has no national accreditation body or standardized licensure requirements for postgraduate training. Jordan has implemented a structured GME system with national exams, continuing medical education (CME), and faculty development initiatives, offering a useful regional model. 9 10 11 12 Saudi Arabia has centralized education quality through the National Commission for Academic Accreditation and Assessment, 13 established in 2005. In contrast, Syria's program standards and educational outcomes remain inconsistent.

Meaningful reform will require the development of an independent national accrediting authority, the introduction of competency-based curricula, national examinations, structured research access, and the integration of CME. These reforms must be aligned with international best practices and tailored to Syria's unique post-conflict context. To this end, the SAMS-led workshops in Damascus and Aleppo in February 2025 brought together over 45 key stakeholders, including directors of teaching hospitals, medical educators, and Syrian diaspora experts, to identify strategic priorities and pathways for reform. Workshop sessions included presentations of pre-workshop survey data and facilitated group discussions to shape actionable recommendations.

Rebuilding Syria's medical education system will depend on coordinated efforts across three interconnected domains: Undergraduate Medical Education (UME), GME, and CME.

Strengthening these pillars is essential to expanding access to high-quality care and training the next generation of health professionals who will serve Syria's recovering population.

### Workshop Overview and Methodology

#### General Objective

To improve GME in teaching hospitals and foster collaboration between the Ministry of Health and the Ministry of Higher Education in Syria.

#### Specific Objectives

- Assess the current status of GME in Syria.
- Identify systemic challenges in delivering clinical training.
- Facilitate knowledge-sharing between local institutions and Syrian experts abroad.
- Develop a phased framework for GME reform.

### Pre-Workshop Survey

A needs assessment pre-workshop survey was developed based on standards and policies from the ACGME and other accrediting councils. A call for participation was then distributed to all deans of medical schools in Syria, as well as directors of teaching hospitals and universities, inviting them to attend one of two in-person workshops, focusing on the following:

- Oversight and governance.
- Accreditation status.
- Faculty-to-resident ratios.
- Curriculum structure.
- Evaluation methods.
- Research access.
- Financial and logistical barriers.

The questions used in the survey are added to Supplement Material S1 (available in the online version only).

### Workshop Details

Two workshops were planned to cover the majority of teaching institutions in Syria, one in the North to cover northern Syria, Aleppo, Hama, and Idlib, and one in the south to cover Damascus, Homs, and Daraa. Both workshops followed the same format, each lasting ∼4 to 5 hours. All discussions and meeting outcomes were documented in formal summary reports to guide future planning and implementation.

- Damascus Workshop: February 23, 2025—21 participants, 10 volunteers.
- Aleppo Workshop: February 25, 2025—24 participants, 4 volunteers.

**Each session included the following:**

- A presentation of the needs assessment survey findings.
- A review of current best practices in GME from the World Health Organization (WHO) and countries with comparable healthcare systems.
- Roundtable discussions that focused on addressing local challenges in medical education. Key takeaways and proposed solutions from each table were compiled into a summary document.
- Participants included directors of teaching hospitals, medical education coordinators from both the Ministry of Health and Higher Education, and SAMS representatives from Turkey and Syria.

## Key Pre-Workshop Survey Findings

### Respondent Demographics and Institutional Representation

A total of **21 individual survey responses** were collected from institutions distributed across **six Syrian governorates** . The institutional geographic breakdown is as follows:

- **Damascus:** 8 responses ( **38.1%** ).
- **Idlib:** 5 responses ( **23.8%** ).
- **Homs:** 2 responses ( **14.3%** ).
- **Hama:** 2 responses ( **9.5%** ).
- **Aleppo:** 3 responses ( **9.5%** ).
- **Rural Damascus:** 1 response ( **4.8%** ).

Each institution listed the full name of the hospital or training center. These included a **wide range of public, private, and conflict-zone hospitals** , including both Syrian Board of Medical Specialties (SBOMS)- and SAMS-affiliated centers:

- Aleppo University Hospital.
- Ibn Al-Nafees Hospital (Damascus).
- Al-Tall National Hospital.
- Pediatric University Hospital (Damascus).
- Al-Shifa Hospital (Afrin).
- Idlib Central Hospital.
- Dana Maternity and Pediatric Hospital.
- Cardiac Surgery Hospital (Damascus).
- Ibn Al-Nafees General Hospital.
- Homs University Hospital.
- Homs National Hospital.
- Maternity and Pediatric Hospital (Idlib).
- Ibn Sina Children's Hospital (Idlib).
- Bab Al-Hawa Hospital.
- Dermatology and Venereology University Hospital (Damascus University).
- General Eye Surgery Hospital.
- SBOMS and SAMS-partnered facilities.

### Authority and Oversight of Training Programs

#### Approval Mechanisms

Institutions identified varying structures of approval:

- Three respondents cited the general manager as the sole approver.
- Two referenced hospital directors.
- Two indicated deans of medical faculties.
- One referred to the University Faculty Council.
- One mentioned the Board of the College of Medicine.
- One noted approval by the SBOMS scientific council and department chairs.
- One said no educational program exists at all.
- One listed both the SBOMS and SAMS, depending on the training stream.
- Others referenced combinations of department heads, scientific deputies, and external coordinators.

#### Training Oversight

Programs are overseen through various structures:

- Lectures and curriculum: Nine institutions had structured monthly lecture schedules for each department.
- Clinical supervision: Seven reported daily or weekly patient rounds, operative supervision, or topic-based updates.
- Scientific monitoring: SAMS and SBOMS had their own councils or faculty tracking training progress.
- Unstructured models: Four responses described “spontaneous,” “traditional,” or “absent” structures.
- Administrative transitions: Two institutions indicated uncertainty due to recent leadership changes.

### Supervision Committees and Meeting Documentation

- 10 institutions (47.6%) had formal training committees.
- Committee composition ranged from 3 to 12+ members:
  - 12 (obstetrics/gynecology) specialists.
  - 13 pediatric specialists.
  - 3 radiology specialists.
  - 2 surgical subspecialists.
- Roles included training coordinators, department heads, hospital administrators, and board leaders.
- Meeting frequency:
  - Weekly: 1 institution.
  - Monthly: 4 institutions.
  - Quarterly: 2 institutions.
  - Annually: 2 institutions.
  - Ad hoc or nonexistent: 2 institutions.
- Documentation: 7 institutions (46.2%) confirmed recording meeting minutes, while 6 did not document.

### Program Size and Training Scope

The size and scope of GME programs varied widely across institutions:

- Number of specialty programs per institution ranged from 1 to 29.
- Number of residents per institution ranged from 13 to 990.

#### Institutional Examples

- One institution reported 23 residency programs with a total of 990 residents (specific specialties not specified).
- Another reported 29 programs and 667 residents, including:
  - A pediatric surgery program with 22 residents and a 6-year training duration.
  - A pediatric department housing 420 residents across 9 pediatric subspecialties.
  - Other departments, such as family medicine, anesthesia, and internal medicine, had between 6 and 40 residents each.
- **Most common residency specialties:**
  - **Core medical and surgical specialties:**
    ▪ General surgery.
    ▪ Obstetrics and gynecology.
    ▪ Pediatrics.
    ▪ Internal medicine.
  - **Other frequently reported specialties:**
    ▪ Vascular surgery.
    ▪ Thoracic surgery.
    ▪ Urology.
    ▪ Pediatric surgery.
    ▪ Ear, nose, and throat (ENT).
    ▪ Maxillofacial surgery.
    ▪ Cosmetic surgery.
    ▪ Burn surgery.

### Salary and Financial Support

- SBOMS trainees: $500/month.
- SAMS trainees: $1000/month.
- Other trainees: salaries ranged approximately between $14 and $47 per month.
- Minimum salary reported: $2/month.
- All 21 institutions confirmed having a clear organizational hierarchy.

### Curriculum and Educational Content

- 57.1% ( *n* = 12) reported having a formal curriculum.
- 42.9% ( *n* = 9) reported no formal or written curriculum.

#### Components Included

- **Theoretical content** : Lectures, journal clubs, topic-based presentations.
- **Practical training** : Hands-on operative participation, simulation sessions, structured task progression.
- **Specific curricula mentioned** :
  - Obstetrics/Gynecology: Logbooks by year, cesarean quotas, core procedures.
  - Dermatology: Structured 4-year plan including cosmetic, oncology, and immunologic diseases.
  - Pediatrics: Year-wise skills checklist for resuscitation, nasogastric tube placement, and IV access.
- **Educational resources used** :
  - Nelson Textbook, European Society of Cardiology (ESC) Guidelines, Braunwald, Tintinalli, UpToDate, PubMed.

### Evidence-Based Medicine

- 10 institutions (47.6%) incorporated evidence-based medicine (EBM) into training.
- Common tools included:
  - UpToDate (6 institutions).
  - PubMed and Medscape (4 institutions)
  - ESC Guidelines, Nelson, individual expert-led sessions.
- The remaining 11 institutions reported no structured EBM education or reliance on traditional lectures only.

### Evaluation and Examinations

- 17 institutions (81%) reported final evaluations.
- Evaluation tools included:
  - Written multiple-choice question (MCQ) exams, objective structured clinical examination (OSCE)-style exams, oral viva, and surgical skills demonstrations.
  - Logbook reviews, weekly quizzes, rotation-based oral assessments.
  - Final board exams either by the SBOMS or internal committees.
- 2 institutions required research presentations or thesis defense.
- 4 institutions (19%) had no structured final exam or depended only on ongoing observation.

### Clinical Procedures and Case Requirements

- 12 institutions (66.7%) tracked clinical procedures.
- Tools used: Logbooks, manual tracking by department heads, Excel checklists, or internal platforms.
- Case minimums by specialty:
  - Pediatrics: Intravenous (IV) access, neonatal cardiopulmonary resuscitation (CPR), cerebrospinal fluid (CSF) tap, nasogastric tube placement, intubation, arterial blood gas sampling.
  - Dermatology: biopsies, excisions, patch tests, cosmetic interventions
  - Surgery: burn debridement, cleft lip/palate repair, flap closure, hernia repair, trauma stabilization, oncology resection.
  - Anesthesiology: intravenous access, intubation, spinal anesthesia, epidural anesthesia, and nerve blocks.
  - General surgery: excision of nevi, suturing of acute and secondary wounds, excision of benign masses, harvesting of autologous grafts, upper and lower eyelid lifts, burn dressing, local flaps, debridement of sacral ulcers, hematoma evacuation, primary and secondary burn interventions, release of contractures (elbow, neck, knee, axilla, fingers), fat grafting, brow lift, flap coverage for large tissue loss, liposuction, tissue expanders, abdominoplasty, facelift, rhinoplasty, breast lift and reduction, gynecomastia treatment, brachioplasty, auricular reconstruction, cleft lip repair, post-mastectomy and post-trauma reconstructive flaps, syndactyly release, polydactyly correction, Fournier's gangrene reconstruction, and assistance in general anesthesia at various levels from fourth to first assistant.

### Workload, Leave, and Logistics

#### Weekly duty hours ranged from

- 20 to 112 hours per week:
  - Common averages: 60–80 hours/week.
  - Several residents reported 3–4 overnight calls/week.
  - Minimum: 20 hours/week in low-volume centers.
  - Maximum: 112 hours/week (including 4+ 24-hour shifts).

#### Leave policies

Annual leave: 14–30 days/year.
Sick leave: 9–15 days/year.
Three institutions reported having no leave or unclear leave rules.

#### Transportation

Out of 21 institutions, 6 (28.6%) provided transportation, while the remaining 15 (71.4%) did not.

### SWOT Analysis of the Pre-Workshop Survey

The leader of the workshops (A.S.) performed a needs assessment using a SWOT (Strengths, Weaknesses, Opportunities, and Threats) model before the visit from Syrian American graduates and then verified the information with attendees in both Aleppo and Damascus workshops, as outlined in Table 1 .

**Table 1:** SWOT analysis of the pre-workshop survey

Strengths	Weaknesses
• The eagerness of trainees to learn • Well-established institutions • Structured/controlled Public support of education	• Variability (or lack of) in quality between different locations • Bureaucracy and regulations • Accessibility and language /culture barriers
Threats	Opportunities
• Local independence • Corruption or incompetence from the leaders of these programs • Lack of funding	• Engagement between Syrian doctors and the diaspora • Availability of Syrian experts inside and outside Syria • Protection of trainees

## Workshops Reports

### Damascus Workshop

#### Challenges in Specialized Medical Education

- Post-war infrastructure damage has significantly impacted the quality of medical education.
- Brain drain due to the forced migration of students and professors.
- Limited access to education and a deteriorating curriculum structure.
- Absence of structured residency training programs, with education heavily relying on individual instructors.
- Lack of standardized admission criteria and training quality leads to inconsistent outcomes.
- Political influence on decision-making, limiting the independence of medical education. Mismatch between the Ministry of Health and the Ministry of Higher Education, leading to varying educational outcomes.

#### Residency Training Challenges

- Financial instability: Resident salaries are insufficient.
- Lack of scientific standards in residency training.
- Limited access to research platforms (e.g., UpToDate and research databases). Research and publication depend on personal efforts, though a research support center has recently been established.
- High dropout rates among residents due to poor training conditions and a lack of qualified faculty.

Need for structured evaluation of training programs and supervisors.

### Aleppo Workshop

#### Challenges in Medical Education

- Limited resources and outdated curricula that lack continuous updates impact the quality of education.
- Absence of accreditation mechanisms affecting global university recognition and standardization.
- Shortage of medical professionals willing to remain in the system due to financial and logistical constraints.
- Insufficient clinical training opportunities and an inequitable distribution of educational resources.
- Lack of a systematic evaluation framework for assessing physicians' competencies and training outcomes.
- Shortage of qualified faculty members and lack of support for educators, affecting training quality.
- Weak English proficiency among students limits access to international research and medical education resources.
- Nursing licensing issue: University nursing graduates are not granted professional practice licenses in hospitals, creating workforce gaps.

Discrepancies in nursing education and certification across different institutions. Mismatch between the number of medical graduates and available GME training spots, causing delays in specialization.

- Lack of standardized certification exams and insufficient flexibility in career pathways for medical graduates.
- Deficiencies in medical education infrastructure, including simulation laboratories and high-speed internet for telemedicine training.
- Limited specialized faculty training programs are reducing the effectiveness of residency training.

### Damascus and Aleppo Workshop Proposed Solutions and Recommendations

Based on the discussions and key points raised in the Damascus and Aleppo Workshop.

#### Advancing GME

**Fig. 1:**
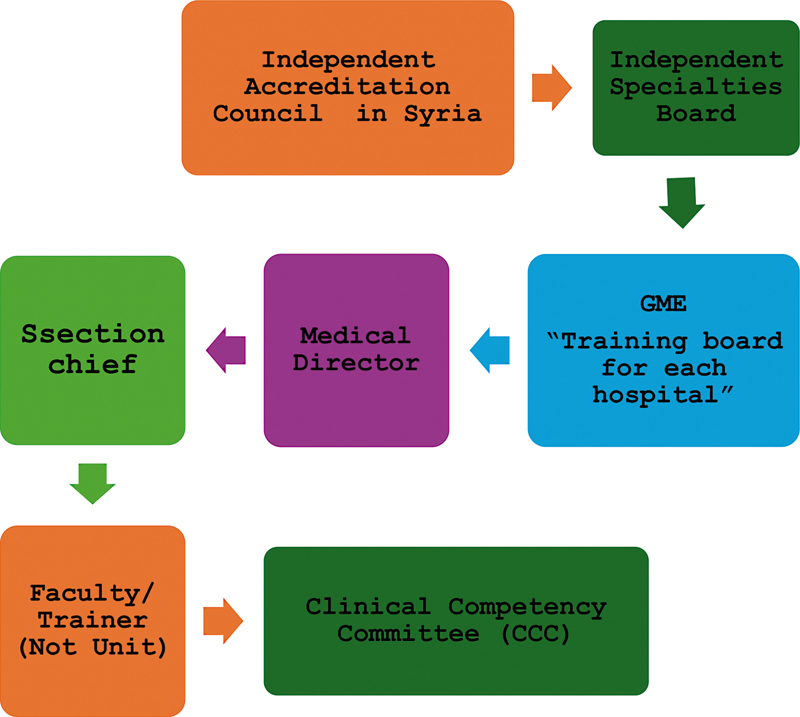
A depiction of the proposed graduate medical education organization structure.

- Updating medical curricula to align with international standards.
- Reevaluating the distribution of medical residents based on the needs of hospitals and healthcare centers.
- Establishing an independent body to assess educational programs and unify medical curricula between the Ministry of Health and the Ministry of Higher Education. Enhancing English-language education without eliminating Arabic by incorporating key lectures in English.
- Below is the organizational chart for GME, which was also discussed during the workshop. This structure is designed to ensure effective oversight and management of residency and fellowship programs ( Fig. 1 ).

#### Improving the Clinical Training Environment

- Revising and assessing the current educational programs in health sciences, such as nursing and allied health professions, to allow the needed healthcare workers to join the workforce in Syria.
- Developing medical education infrastructure in teaching hospitals, including lecture halls, surgical theaters, simulation, and anatomy laboratories.
- Conducting periodic evaluations of teaching hospitals to ensure quality training.
- Requiring hospitals to establish clear training plans that outline their need for residents and specialists.
- Implementing a “resident rotation” model between different hospitals (public or private) to enhance training opportunities

#### Strengthening the Role of Faculty and Supervisors

- Defining the role of academic supervisors in medical departments with clear job descriptions.
- Setting criteria for selecting faculty members and medical educators, requiring at least 10 years of experience.
- Limiting the number of trainees per supervisor based on specific guidelines (suggested ratio: three postgraduate students per academic professor and five trainees per clinical professor per year).

#### Establishing Independent Regulatory Bodies for Quality Assurance

- Creating a national body to assess and accredit training programs, composed of academics and medical education experts.
- Introducing a medical education regulatory body with two branches:
  - Scientific branch—Responsible for curriculum development and program enhancements.
  - Administrative branch—Responsible for monitoring implementation and ensuring quality.
- Merging the Ministries of Health, Higher Education, and Defense under a unified independent authority to train specialists based on local needs.

#### Strategic Plans for Medical Education Development

##### Short-Term Plans (1–3 Years)

- Appointing a director for medical education in each teaching hospital to oversee GME specialty programs.
- Assessing the competency of the current graduating class and considering an extension with salary for those who need additional training time.
- Expanding online medical education platforms, both synchronous and asynchronous.
- Conducting training programs for faculty members to enhance teaching skills. Launching specialized training programs to support continuous medical education.
- Adjusting the number of available GME spots for the next academic year based on healthcare system demands.

##### Medium-Term Plans (3–5 Years)

- Reducing the number of trainees per program to improve quality based on available volume and availability of qualified faculty.
- Providing financial incentives to attract and retain medical professionals.
- Introducing specialized training courses to improve residents' skills.

##### Long-Term Plans (+5 years)

- Support Syrian universities to achieve international recognition.
- Enhancing student engagement for better educational outcomes.
- Boosting medical research and ensuring access to global academic resources.

### Strengthening Oversight and Evaluation Mechanisms

- Ensuring proper documentation of meeting minutes to track progress in medical education reforms.
- Establishing an independent evaluation committee for regular quality assessments of training programs.
- Adopting other successful training systems, such as the United States (Accreditation Council for Graduate Medical Education [ACGME]), or the Canadian model for clinical skill evaluation, ensures standardized assessment criteria.

### Additional Key Discussion Points

- Reassessing the nationwide open-enrollment policy to align with the actual demands of the healthcare sector.
- Establishing an independent Syrian council for GME accreditation, composed of medical education experts inside and outside Syria, to oversee all accreditation processes.
- Bridging the gap between medical schools and specialty training spots by introducing master's programs and research positions (1–2 years) in universities without clinical specialties.
- Launching a 1-year rural medicine training program after medical school to prepare general practitioners for underserved areas.
- Allowing residents to rotate in private hospitals as part of their training to gain diverse clinical experience under qualified teaching faculty.

### Comparison Between the Damascus and Aleppo Workshops

- The main differences were that the attendees were from different locations (including Northwest Syria and Hama, which have similar experiences compared with Aleppo; however, slightly different experiences). In addition, dental students were in attendance in Aleppo and not in Damascus.
- Both workshops emphasized the need to develop GME and implement standardized curricula.
- Both highlighted the importance of independent regulatory bodies for monitoring and accrediting medical training programs.

## Conclusion

The Damascus and Aleppo GME Workshops provided a comprehensive assessment of the challenges facing medical education in Syria. The discussions emphasized the urgent need for reform to improve the quality, oversight, and financial sustainability of residency training programs. The proposals outlined in this white paper are not only feasible but essential for restoring trust, quality, and efficiency in Syria's healthcare system. Collaboration between ministries, universities, and international experts will be key. By embracing innovation, standardization, and global alignment, Syria can create a resilient, modern medical education system capable of meeting national and international health needs.

## Key Next Steps

1. Initiating discussions with government stakeholders to advocate for an independent accreditation council.
2. Developing faculty training programs to enhance teaching quality.
3. Expanding research opportunities and access to EBM resources.
4. Implementing structural changes in hospital-based medical education to align with international best practices.
5. Expanding upon these findings by addressing other critical aspects of medical education in Syria, including UME and CME. Additionally, we plan to revisit this topic to assess the implementation and impact of the recommended interventions through continued stakeholder engagement and follow-up analysis.

By adopting these strategic recommendations, GME can strengthen its medical education system, enhance physician training, and improve patient care outcomes.

## Workshop Presentations

Two regional workshops were conducted to cover the majority of Syria's teaching institutions:

1. **Damascus Workshop:** February 23, 2025—21 participants, 10 volunteers.
2. **Aleppo Workshop:** February 25, 2025—24 participants, 4 volunteers.

Participants included directors of teaching hospitals, medical education coordinators from both the Ministry of Health and the Ministry of Higher Education, and SAMS representatives from Turkey and Syria.
