# Supplementary material for: Reforming Graduate Medical Education in Syria: A Strategic Framework for Post-Conflict Recovery
Source: Avicenna J Med. 2025 Sep 26;15(3):123–30. doi: 10.1055/s-0045-1811693 (PMC12520756; doi:10.1055/s-0045-1811693)
Supplement: Supplementary file 1 — Supplementary Material [file 10-1055-s-0045-1811693-s250044.pdf]

## Supplementary Material S1 Pre-assessment of graduate medical education in Syria

What is the name of your hospital?
Who is responsible for the approval of the graduate medical education (GME) program at your hospital (e.g., director, chair(s), faculty dean, or someone else)?
How does the responsible person demonstrate oversight over training programs?
Do you have a dedicated committee to oversee training at your hospital? If so, how many and who are they (e.g., program directors)? If so, how many times do they meet per year, and do they give minutes?
How much do residents get paid as a stipend per year?
Do you have an organizational chart?
What is the recognition requirement of the specialty training program?
How long has this hospital been accredited to train physicians and which program first?
How many GME specialty programs do you currently have? And how many total trainees do you have currently?
Do you have a person in charge of oversight of all training programs at your hospital? If so, what is their title?
List all programs and the number of trainees, how many years the training is for, and how many per year (e.g., general surgery: 5 years, and each year takes two trainees).
Do you have a curriculum for each training program?
If so, please describe the curriculum.
Do you teach evidence-based medicine? If so, what are the sources?
How do you evaluate trainees?
How many teaching faculty do you have for each training program?
What is the faculty:trainee ratio in each program in your hospital?
What professional development do you have for your faculty (e.g., any educational workshops or continued medical education)?
Do you have research at your hospital to support medical education trainees?
Do you have access to medical literature? Please specify.
What opportunities should on-training physicians or faculty members have for raising concerns and providing feedback confidentially?
Do you monitor the work hours of your trainees? What are the average hours per week for medical and for surgical trainees?
Do you provide transportation to your trainees?
How many weeks do trainees receive time off (vacation, sick leave)?
Do you require a summative final evaluation before graduation? If so, what does the form include?

**For medical specialists:**

Do you track procedures to be eligible to graduate (e.g., intubation or endoscopy procedures)?
If so, list these procedures and the minimum number requirement.
What system do you use to track these procedures?

**For surgical specialties:**

Do you track the number of care per trainee?
Please list all the required cases to graduate.
What system do you use to track these case logs?
Is there a minimum number of cases required to be eligible to graduate? If so, add that to the above list?

 © 2025. The Author(s).
